# Formulation of 1% α-mangostin in orabase gel induces apoptosis in oral squamous cell carcinoma

**DOI:** 10.1186/s12906-024-04450-0

**Published:** 2024-07-20

**Authors:** Wipawee Nittayananta, Teerapol Srichana, Jureeporn Chuerduangphui, Ekarat Hitakomate, Kesinee Netsomboon

**Affiliations:** 1 Faculty of Dentistry, Thammasat University, Pathum Thani, Thailand; 2 Drug Delivery System Excellence Center, Faculty of Pharmaceutical Sciences, Prince of Songkla University, Hat Yai, Songkhla, Thailand; 3 Department of Pharmaceutical Technology, Faculty of Pharmaceutical Sciences, Prince of Songkla University, Hat Yai, Songkhla Thailand; 4 Department of Microbiology, Faculty of Science, Kasetsart University, Bangkok, Thailand; 5 Department of Pharmaceutics and Industrial Pharmacy, Faculty of Pharmacy, Thammasat University, Pathum Thani, Thailand

**Keywords:** Apoptosis, Oral cancer, α-mangostin, Oral gel, Oral squamous cell carcinoma

## Abstract

**Background:**

Plant-derived compounds have chemopreventive properties to be used as alternative medicine. Pericarp of Mangosteen (*Garcinia mangostana* Linn.), a tropical fruit in Southeast Asia contains a phytochemical *α*-mangostin (*α*-MG) that demonstrates potent anticancer effects against various types of cancer. *α*-MG has been reported to be the most effective agent in human cancer cell lines. The objectives of this study were to develop oral gel formulations containing α-MG and determine their (1) anticancer activity, (2) anti-HPV-16 and antimicrobial activities, (3) nitric oxide (NO) inhibitory activity, and (4) wound healing effect.

**Methods:**

Formulations of oral gel containing α-MG were developed. Anticancer activity on SCC-25 was assessed. Apoptotic induction was determined using flow cytometry technique. Antiviral activity against HPV-16 pseudovirus and antimicrobial activity against *S. mutans, P. gingivalis* and *C. albicans* were investigated. NO inhibition was carried out. Fibroblast cell migration was determined by in vitro scratch assay.

**Results:**

The formulation of 1% α-MG in orabase gel demonstrated anticancer activity by promoting apoptosis in SCC-25. The induction of apoptotic activity was dose dependent with pronounced effect in late apoptosis. The formulation appeared to reduce cell viability of oral keratinocytes (OKC). At CC_50_ it showed an inhibition against HPV-16 pseudovirus infection. The formulation had no antimicrobial activity against *S. mutans, P. gingivalis* and *C. albicans.* No significant NO inhibitory activity and wound healing effects were found.

**Conclusions:**

1% α-MG in orabase gel exhibited anticancer activity by inducing apoptosis although low level of cytotoxicity observed in OKC was present. The appropriate carrier for novel nano-particles targeting cancer cells should be further investigated.

## Introduction

Although diagnosis and treatment of oral cancer become advancing nowadays, it is still a significant oral health problem worldwide. Among this, oral squamous cell carcinoma (OSCC) is the most common accounted of more than 90% of all oral cancers [1]. Over the past two decades, the overall five-year survival rate of oral cancer has remained at ∼60% [2]. The incidence of OSCC is mostly found in people older than 65 years old. However, incidence in adolescents is rising every year. OSCC is caused by tobacco, alcohol, betel nut chewing, and infection by human papillomavirus (HPV) [3]. It may be preceded by the lesions called oral potentially malignant disorders (OPMD) [4]. Thus, early detection and treatment to prevent progression of the lesion may reduce the mortality rate and bring a better prognosis [5].

Plant-derived compounds have chemopreventive properties which may be used as an alternative method in preventing or delaying tumor progression [3, 6]. Mangosteen (*Garcinia mangostana* Linn.) is the tropical fruit grows in Southeast Asia. Its pericarp contains a variety of phytochemicals used as traditional medicines [4]. Among these, xanthones are one of the phytochemical groups in the mangosteen peel that show various biological activities such as anticancer, anti-inflammatory, antimicrobial, antiallergy, antioxidant, and cardioprotective properties [7]. The mangosteen pericarp-derived xanthones, *α*-mangostin (*α*-MG) is the most abundant phytochemical that demonstrates potent anticancer effects against various types of cancer [8–11]. Due to its therapeutic benefits including anticancer properties, *α*-MG has been reported to be the most effective agent in human cancer cell lines [12, 13].

Although early detection and prevention of malignant transformation of the OPMD lesions is an appropriate approach, oral products to be used for such purpose are limited. Because *α*-MG has been reported to serve as an anticancer agent against different types of cancer cells including OSCC [3], we aimed to develop formulations of oral gel containing the compound. We hypothesized that the α-MG oral gel possesses anticancer, anti-inflammatory and antimicrobial activities and enhance wound healing and that it could potentially prevent the progression of OPMD lesions to OSCC. Our previous study reported that mucoadhesive film containing *α*-MG inhibited growth of SCC-25 [14]. However, its related biological mechanisms have not been well established. Therefore, the purpose of this in vitro study was to formulate the oral gel containing α-MG along with other edible ingredients and tested for its in vitro (1) anticancer and apoptotic activities; (2) anti-HPV-16 and antimicrobial activities; (3) NO inhibitory activity, and (4) wound healing effect.

## Materials and methods

### α-mangostin

Mangosteen peel extract containing 75% α-MG used in this study was purchased from Kasetsart University, Bangkok, Thailand (food grade).

### Preparation of oral gel containing α-mangostin

Sodium carboxymethylcellulose, pectin and gelatin (MW 50,000) were obtained from PC Drugs, Bangkok, Thailand. Plastibase (polyethylene MW 21,000 5% and liquid paraffin 95%) was prepared according to Glasshouse Pharmaceuticals limited, Canada. Other chemicals used were of pharmaceutical and analytical grades. Oral gel containing active ingredients of 75% α-MG was prepared in two different formulations:

Formulation 1: α-MG oral gel contained 1% wt carbopol 940, 3% wt sodium carboxymethylcellulose, 13% wt polyethylene glycol (PEG 400) and 83% water, respectively. Gel base was prepared by thoroughly mixing of hydrated polymeric material stated previously. Afterwards, PEG 400 and paraben concentrate were added to the mixture. *α*-MG was incorporated to the gel base formulation to give final concentration of 0.4 mg per g of gel base.

Formulation 2: 1% α-MG in orabase gel was prepared by mixing equal weight ratio of adhesive base (sodium carboxymethylcellulose: pectin: gelatin) and plastibase. The adhesive base was prepared by first dispersing gelatin 16.6 g in 50 ml of hot water at 85 °C. After gelatin solution cooling down, sodium carboxymethylcellulose and pectin were added with continuous stirring. The compound of Mangosteen peel extract 1 g was levigated with orabase gel 99 g by geometric dilution to obtain 1%α-MG in orabase gel.

## Culture conditions

### Cell culture

#### Squamous cell carcinoma cell line

The Homo sapiens tongue squamous cell carcinoma cell line SCC-25 was purchased from ATCC and maintained in cultures system according to the previously described procedures [14]. Briefly, cells were cultured in Dulbecco’s Modified Eagle Medium (DMEM; Gibco, NY, USA) supplemented with 15% fetal bovine serum (FBS: Gibco, NY, USA) and 100 U/ml antibiotic–antimycotic (Gibco, NY, USA). The cells were incubated under 37 °C and 5% CO_2_ atmosphere until reaching 80% confluent then were trypsinized with 0.25% trypsin-EDTA (Gibco, NY, USA). To examine cell viability, cells were stained with 0.4% trypan blue (Gibco, NY, USA) and visualized under the light microscope.

#### Oral keratinocyte cell line

The primary oral keratinocyte (OKC) cell line was provided by the Faculty of Dentistry, Chulalongkorn University, Bangkok, Thailand. Cells were maintained in culture system according to the previously described protocol [15]. Briefly, the cell line was grown in Defined Keratinocyte Serum Free Media (Gibco, NY, USA), supplemented with Keratinocyte Growth factors (Gibco, NY, USA) and 100 U/ml antibiotic–antimycotic (Gibco, NY, USA). The cells were incubated under 37 °C and 5% CO_2_ atmosphere until reaching 80% confluent then were trypsinized with 0.25% trypsin-EDTA (Gibco, NY, USA) and inactivated enzyme activity by 10% FBS (Gibco, NY, USA) in 1x PBS. Cell viability was examined using 0.4% trypan blue (Gibco, NY, USA) under the light microscope.

#### Human embryonic kidney 293FT cell line

Human embryonic kidney (HEK) 293FT cell line was purchased from Invitrogen company (Carlsbad, CA, USA). Cells were maintained in DMEM (Gibco®, USA) supplemented with 10% FBS and antibiotics (gentamycin, penicillin G, streptomycin and fungizole). The cells were incubated at 37ºC in a 5% CO_2_ incubator and subcultured at 80–90% confluence.

#### Human gingival fibroblast cell line and mouse monocyte like macrophage cell line

Human gingival fibroblast (HGF) cell line provided by the Faculty of Dentistry, Prince of Songkla University, Thailand, and mouse monocyte like macrophage cell line (RAW264.7, ATCC TIB-71, USA) were grown to produce a new single cell suspension for further incubation as previously described [16]. Briefly, the cells were cultured in Dulbecco’s Modified Eagle Medium (DMEM, Gibco®, USA) containing 10% fetal bovine serum (FBS, Gibco®, USA) and antibiotics (100U/ml penicillin/streptomycin, Gibco®, USA) under 5% CO_2_ at 37 ºC. The media were changed every other day. When the cells reached confluence, they were harvested using 0.25% trypsin-EDTA (Gibco®, USA), followed by the addition of fresh culture medium to create a new single cell suspension for further incubation.

The mouse monocyte/macrophage cell line (RAW264.7, ATCC TIB-71, USA) was cultured in DMEM (Gibco®, USA) supplemented with 10% FBS (Gibco®, USA), 100 U/ml penicillin/streptomycin (Gibco®, USA). The cells were incubated at 37ºC in a 5% CO_2_ incubator and the media was changed every 2 days. They were harvested by gentle rocking, followed by the addition of fresh culture medium to create a new single cell suspension for further incubation.

### Cell viability assay

Cytotoxicity was determined by the MTT assay as previously described [14]. Briefly, the SCC-25, oral keratinocytes, HGF, RAW264.7 and 293FT cell lines were maintained in culture after being harvested with 0.25% trypsin-EDTA and diluted to a suspension in a fresh medium. The cells were seeded in 96-well plates with 5 × 10^4^ cells/well (for SCC-25, HGF and RAW264.7 cell lines) 3 × 10^4^ cells/well (for OKC), and 1 × 10^4^ (for 293FT cell line) and allowed to adhere at 37 °C for 24 h. Next, the medium was replaced with various concentrations of the compound and the formulations containing α-MG and was then incubated for 24 h for SCC-25, OKC and RAW264.7 and 48 h for 293FT. Ten microliter MTT solution (5 mg/ml in PBS) was added to 96-well plates. After a 2 h incubation, the medium was removed, and DMSO (200 µl) was added to each well to dissolve the formazan crystals and then measured with a microplate reader at 540–570 nm (Multiskan™ FC; Thermo Fisher Scientific, Waltham, MA, USA). The test samples were considered cytotoxic when the optical density (OD) of the sample-treated group was less than 80% of that in the control group. Cell viability was calculated using the following equation:

$$\%\text{Cell\,viability} = [\text{OD}_{\text{sample}}/\text{OD}_\text{control}] \times 100$$

### Apoptosis assay

The SCC-25 cells (5 × 10^5^ cells) were cultured overnight in a 25 cm^2^ filter flask. Prior to harvesting cells by trypsinization, cells were exposed to α-MG orabase gel at the IC_10_ (2.0 mg/ml), IC_25_ (3.2 mg/ml) and IC_50_ (5.3 mg/ml) concentrations for 48 h.The cells were then stained with the apoptotic protein marker phosphatidylserine and intracellular DNA using the fluorescence dye according to the manufacturer’s protocol (BD Pharmingen™ FITC Annexin V Apoptosis. Detection Kit, BD Biosciences, CA, USA). The numbers of both living and apoptotic (early and late) cells were examined by FACSAriaIII flow cytometer (BD. Biosciences, CA, USA) at 275–285 and 310–330 energy voltages for FITC and PI, respectively. The data analyses were performed on BD FACSDiva. The independent experiments were conducted in triplicate.

### Antiviral activity against HPV-16 pseudovirus infection in pre-attachment and adsorption steps

#### HPV-16 pseudovirus production

The 293FT cells were seeded and co-transfected with p16SheLL (6 µg) and pfwB (6 µg) plasmids as previously described [14]. In brief, the cells were seeded in a 25 cm^2^ culture flask at 3 × 10^5^ cells/flask and maintained for 4 days. They were co-transfected with p16SheLL (6 µg) and pfwB (6 µg) plasmids kindly provided by John T. Schiller (Laboratory of Cellular Oncology, Bethesda, MD, USA) using Lipofectamine 2000 (Invitrogen, Carlsbad, CA, USA) for 6 h. Two days post-transfection, the transfected cells were harvested and lysed in lysis buffer containing 0.5% Brij 58 (Sigma-Aldrich, St. Louis, MO, USA), 0.2% RNase A (bovine pancreas, Sigma Chemical Company, St. Louis, MO, USA), 9.5 mM MgCl_2_ in PBS. The lysed cells were incubated at 37 °C for 24 h and then chilled on ice for 5 min and centrifuged at 1,500 rpm for 5 min to collect the supernatant containing the pseudovirus used in the experiments.

#### Determination of anti-HPV-16 pseudovirus infection in pre-attachment step

The 293FT cells were seeded in 96-well plate at a density of 3 × 10^3^cells/well and maintained in complete medium for 1 day. HPV-16 pseudovirus at multiplicity of infection (MOI) at 0.05 was mixed with the formulation of 1% α-MG in orabase gel at CC_20_ and CC_50_, and incubated at 37 °C for 1 h. Heparin (400 µM) derived from porcine intestinal mucosa (Sigma-Aldrich, Saint Louis, MO, USA) was used as a positive control. Subsequently, the mixture was added to 293FT cells for 4 h and then was removed and maintained in complete medium for 48 h. The cells were harvested and washed by additional trypsin and PBS respectively. Twenty microliter of cell suspension in complete medium was added to hemacytometer. Total cells and green fluorescent cells in the same area were counted under the microscope (Olympus BX51 fluorescent microscope, Japan). Percentage of inhibition was calculated from green fluorescent relative to total cells, and then compared with the untreated.

#### Determination of anti-HPV-16 pseudovirus infection in adsorption step

The 293FT cells were seeded 3 × 10^3^cells/well in 96-well plate and maintained in complete medium for 1 day. HPV-16 pseudovirus (MOI 0.05) was added to the cells and then incubated at 20 °C for 2 h. After the pseudovirus was removed, the 1% α-MG in orabase gel at CC_20_ and CC_50_ mixed in complete medium was added to the cells and incubated for 48 h. Heparin (400 µM) was used as a positive control. The cells were harvested and washed by additional trypsin and phosphate buffer saline (PBS), respectively. Total cells and green fluorescent cells in the same area were counted under the microscope. Percentage of inhibition was calculated as mentioned above.

### Antimicrobial activity against common oral pathogens

#### Microbial growth condition and inoculum preparation

*S. mutans* (ATCC 25175), *P. gingivalis* (ATCC 33277) and *C. albicans* (ATCC 90028) were cultured in appropriate broth as previously described (16). Briefly, *S. mutans* (ATCC 25175) was cultured on brain heart infusion broth (BHI) and incubated at 37 °C for 18–24 h. *P. gingivalis* (ATCC 33277) was cultured in tryptic soy broth (TSB) supplemented with hemin (5 µg/ml) and vitamin K_1_ (1 µg/ml) and incubated under anaerobic conditions maintained in an anaerobic jar containing a gas-pak microbiology Anaerocult (Merck, Darmstadt, Germany) at 37 °C for 48–72 h prior to use. *C. albican*s (ATCC 90028) was cultured in Sabouraud dextrose broth (SDB) and incubated at 35 °C for 24 h.

### Antimicrobial activity assay

#### The minimal growth inhibition concentration (MIC)

Samples of 1% α-MG in orabase gel were tested against *S. mutans, P. gingivalis* and *C. albican*s using broth microdilution method for their inhibitory activity [17] as previously mentioned [16]. In brief, 100 µl of the serially diluted samples (from 234 –3.66 µg/ml) were added to each well plate and added ten microliters of microbial suspension at a starting optical density at 10^5^ CFU/ml to the sample well plates. The microtiter plates were incubated in the appropriate conditions for each microbial mentioned earlier. Each sample was assayed using four replicates. After the incubation period, 30 µl 0.02% of resazurin sodium salt dissolved in phosphate buffer saline (PBS) was added to each well. The plates were further incubated for 3 h and bacterial growth was indicated by the pink color. The MIC value is the lowest sample concentration at which no pink color observed, i.e., no bacterial growth.

#### The minimal bactericidal concentration (MBC)

MBC was determined by sub-culturing the samples at the MIC or lower cocentration on freshly prepared culture agar plates. After incubation i sutiable conditions for each organism, the MBC value is the lowest concentration with no single bacterial colony.

### Measurement of nitric oxide (NO) production

Nitric oxide produced by RAW 267.4 cells was measured after the cells were exposed with various concentrations of the 1% α-MG in orabase gel (100 µl) or 1 µg/ml of the lipopolysaccharide from *E.coli* (LPS, Sigma-Aldrich) as a positive control as previously described [16]. Briefly, the cells were seeded at a concentration of 1 × 10^6^ cell/ml into 96 well plates in complete medium. After a 24 h incubation, 100 µl of the samples of 1% α-MG in orabase gel at various concentrations or 1 µg/ml of the lipopolysaccharide from *E.coli* (LPS, Sigma-Aldrich) as a positive control in fresh medium, was added to each well. Wells with media only served as negative controls. The Griess reaction assay was used to determine nitric oxide (NO) content in the cell supernatants. Briefly, 100 µl Griess reagent (1% sulfanilamide [Sigma-Aldrich]) in 2.5% phosphoric acid and 0.1% N-(1-naphthyl) ethylenediaminedihydrochloride (Sigma-Aldrich) was mixed with an equal volume of culture media. In this assay, a pink solution indicates a positive result, while a yellow solution indicates a negative result. Based on a standard curve of NaNO_2_, the quantity of NO was determined by measuring the absorbance at 450 nm using a microplate reader. The amount of NO was determined by measuring the absorbance at 450 nm using a microplate reader using a standard curve.

The % NO inhibition was calculated from the following equations.

$$\text{NO\,inhibition} (\%)=((\text{A}-\text{B})/(\text{A}-\text{C}))\times100$$

Where A is absorbance of LPS.

B is absorbance of LPS with sample.

C is absorbance of negative control.

### In vitro scratch assay

Human gingival fibroblast cells were seeded in complete medium with and without 1% α-MG in orabase gel (29.20 mg/ml) and a linear scratch was produced with a sterile pipette tip as previously described [14]. In brief, the cells were seeded into 6-well plates at a density of 1 × 10^6^ cells/well. A linear scratch was generated with a sterile pipette tip in the monolayer when it reached confluence. Cellular debris was removed by washing three times with 3 ml PBS and replaced with 2 ml complete medium containing with 1% α-MG in orabase gel (29.20 mg/ml), while complete medium without the gel served as a negative control. Photographs were taken at a 10x magnification using a microphotograph on day 0, then the plates were incubated at 37 °C with 5% CO_2_ and images were obtained at days 1, 2 and 3. The images acquired for each sample were further analyzed quantitatively using computing software (ImageJ, National Institute of Mental Health, Maryland, USA). Microphotographic images were taken at a 10x magnification using a microphotograph on day 0, day 1, day 2, day 3 and quantitatively analyzed by imaging software (ImageJ, National Institute of Mental Health, Maryland, USA). The distance of each scratch closure was determined by comparing the images from day 0 to 2 as previously described [14]. Two scratches were made in each well (left and right) and six points were considered per scratch. The average of the left scratch and right scratch were taken separately. The percentage migration was calculated for the left scratch and then the right scratch using the following formula:

$$\%\text{Migration\,rate}=\frac{\begin{aligned}&\text{average\, distance\, between\, scratch\, day\, 0}\\&-\text{average\, distance\, between\, scratch\, day}\, 1 \end{aligned}}{\text{average distance between scratch day}\,0}$$

### Statistical analysis

Statistical analyses were performed using Prism5 software (GraphPad, San Diego, CA, USA). All the data were expressed as mean ± SEM (standard error of the mean) from three independent experiments. Comparisons between untreated and treated groups were investigated by Student’s t-tests. Where there are more than two group comparison, One-way ANOVA was used, followed by multiple comparison test to determine statistically significant changes. The symbols *, ** and *** are denoted as statistically significant differences (*P* < 0.05, 0.01 and 0.001, respectively).

## Results

### Physical properties of oral gel containing α-mangostin

Loading capacity of gel base in α-MG orabase gel was only 1% in fact it can be higher than 1–5%. There was no sign of physical instability over 6 months. The orabase gel was yellow straw semisolid with mucoadhesive properties. The pH of the gel was around 6.5. After the freeze-thaw cycle and long-term stability for 6 months the 1% α-MG in orabase gel was stable.

### Cytotoxicity of α-mangostin

**Fig. 1:**
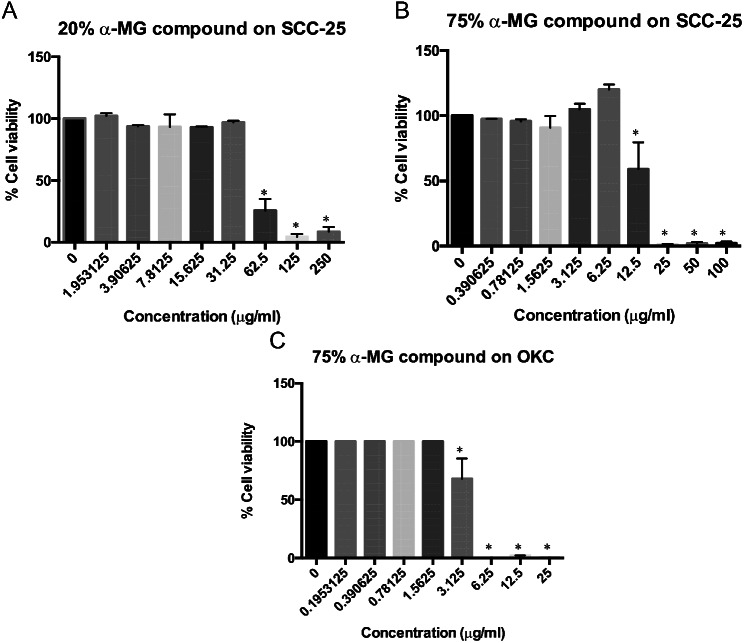
Effects of α-MG compound on viability of SCC-25 and oral keratinocyte cell lines (OKC). Viability of SCC-25 cell line in response to Mangosteen peel extract containing 20% vs. 75% α-MG was determined by MTT assay. Cytotoxic effects were observed at the concentration 62.50 µg /ml and 12.50 µg /ml of 20% and 75% of α-MG with the IC_50_ of 53.50 µg/ml and 20.16 µg/ml, respectively (**A** and **B**). Viability of OKC in response to the compound containing 75% α-MG was also assessed by MTT assay. Cytotoxic effects were observed when the concentration was 3.13 µg /ml with the IC_50_ of 3.30 µg/ml (**C**). Asterisk (*) indicates statistically significant differences of % cell viability from that of untreated cells at p-value < 0.05

Cytotoxicity of Mangosteen peel extract containing 20% and 75% of α-MG on SCC-25 and OKC was determined by MTT assay. The results showed cytotoxic effects on the cell viability of SCC-25 when the concentration of the extract with 20% and 75% of α-MG was 62.50 µg **/**ml (IC_50_ of 53.50 µg/ml) and 12.50 µg **/**ml (IC_50_ of 20.16 µg/ml), respectively. An increase in concentration of the compound led to a decrease in cell viability, and the toxicity on SCC-25 was observed at the concentration of > 31.25 µg/ml for 20% of α-MG, and > 6.25 µg/ml for 75% of α-MG (Fig. 1A and B). Cytotoxicity of Mangosteen peel extract containing 75% of α-MG was also observed on OKC. The compound affected the cell viability of OKC when the concentration of the extract was 3.13 µg **/**ml (IC_50_ of 3.30 µg/ml) (Fig. 1C).

### Cytotoxicity of oral gel containing α-mangostin

**Fig. 2:**
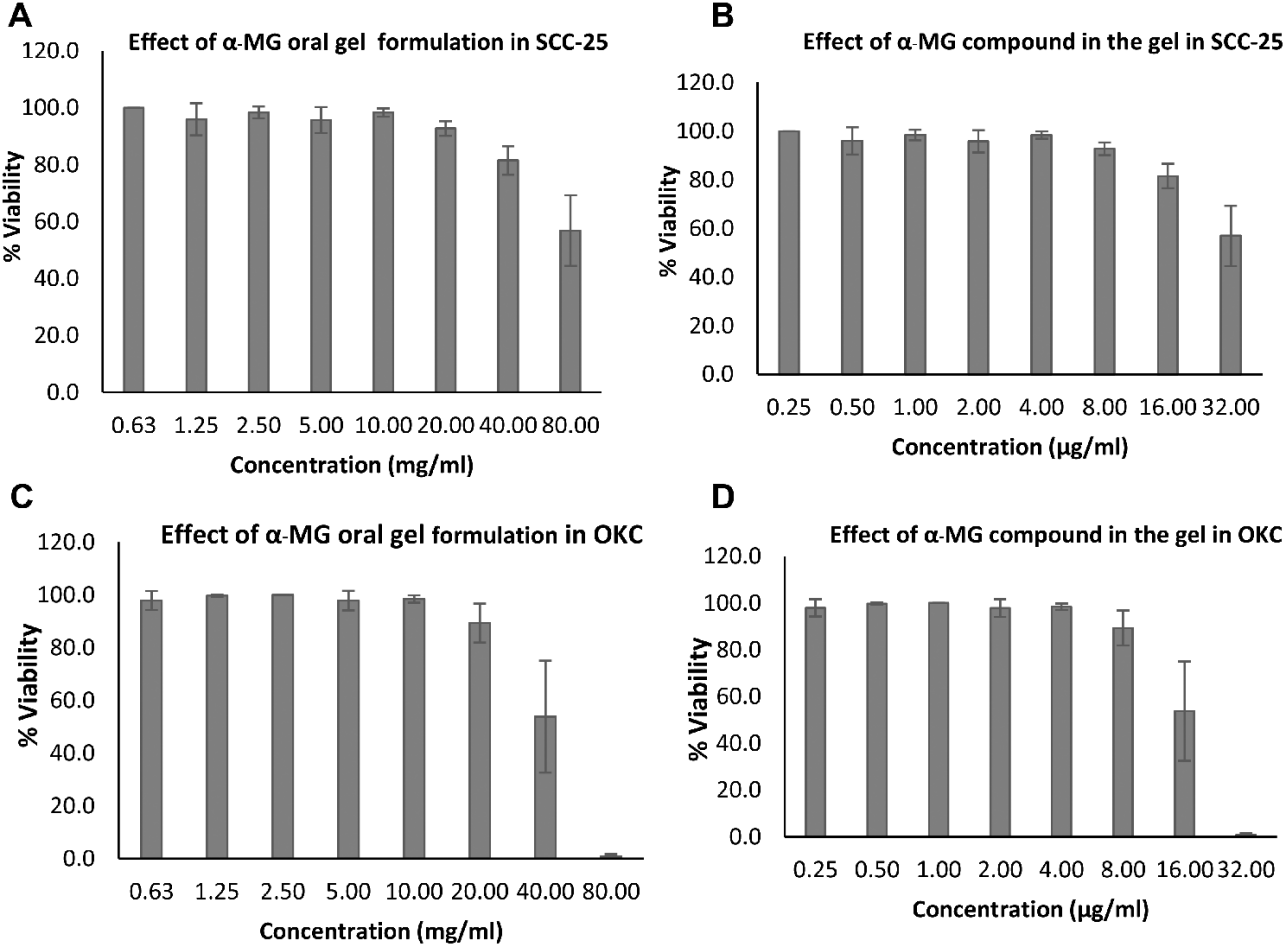
Effects of α-MG oral gel (Formulation 1) on viability of SCC-25 cell line and oral keratinocytes (OKC). Viability of SCC-25 cell line and OKC in response to the formulation of α-MG oral gel was determined by MTT assay. Cytotoxic effects of the gel on the viability of SCC-25 was noted when the concentration of the gel was 80 mg/ml (IC_50_ of 43.77 mg/ml) and with the concentration of the compound in the gel was 32 µg/ml (IC_50_ of 16.45 µg/ml) (**A** and **B**). At those concentrations, the gel also affected the growth of OKC with the IC_50_ of 59.63 mg/ml and 28.00 µg/ml, respectively (**C** and **D**)

**Fig. 3:**
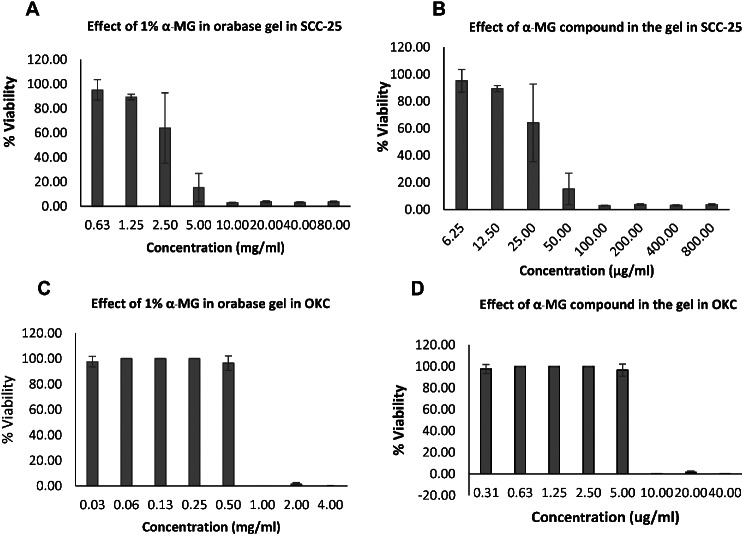
Effects of 1% α-MG in orabase gel (Formulation 2) on viability of SCC-25 cell line. Viability of SCC-25 cell line and OKC in response to the formulation of 1% α-MG in orabase gel was determined by MTT assay. Cytotoxic effects of the gel on the viability of SCC-25 was noted when the concentration of the gel was 5 mg/ml (IC_50_ of 5.33 mg/ml) and with the concentration of the compound in the gel was 50.00 µg/ml (IC_50_ of 53.22 µg/ml) (**A** and **B**). The gel showed cytotoxic effects on the cell viability of OKC when the concentration of the gel was 1 mg/ml (IC_50_ of 0.66 mg/ml) and with the concentration of the compound in the gel was 10.00 µg/ml (IC_50_ of 6.55 µg/ml) (**C** and **D**)

Cytotoxicity of the two formulations of oral gel containing α-MG on SCC-25 and OKC was determined by MTT assay. The formulation 1 (α-MG oral gel) showed cytotoxic effects on the cell viability of SCC-25 when the concentration of the gel was 80 mg**/**ml (IC_50_ of 43.77 mg/ml) and with the concentration of the compound in the gel was 32 µg/ml (IC_50_ of 16.45 µg/ml) (Fig. 2A and B). Effects on the cell growth of OKC were observed with the IC_50_ of 59.63 mg/ml and 28.00 µg/ml, respectively (Fig. 2C and D). The formulation 2 (1% α-MG in orabase gel) showed cytotoxic effects on the cell viability of SCC-25 when the concentration of the gel was 5 mg**/**ml (IC_50_ of 5.33 mg/ml) and with the concentration of the compound in the gel was 50.00 µg/ml (IC_50_ of 53.22 µg/ml) (Fig. 3A and B). Effects on the cell viability of OKC were noted when the concentration of the gel was 1 mg**/**ml (IC_50_ of 0.66 mg/ml) and with the concentration of the compound in the gel was 10.00 µg/ml (IC_50_ of 6.55 µg/ml) (Fig. 3C and D).

### Apoptotic activity of oral gel containing α-mangostin in oral cancer cell line SCC-25

**Fig. 4:**
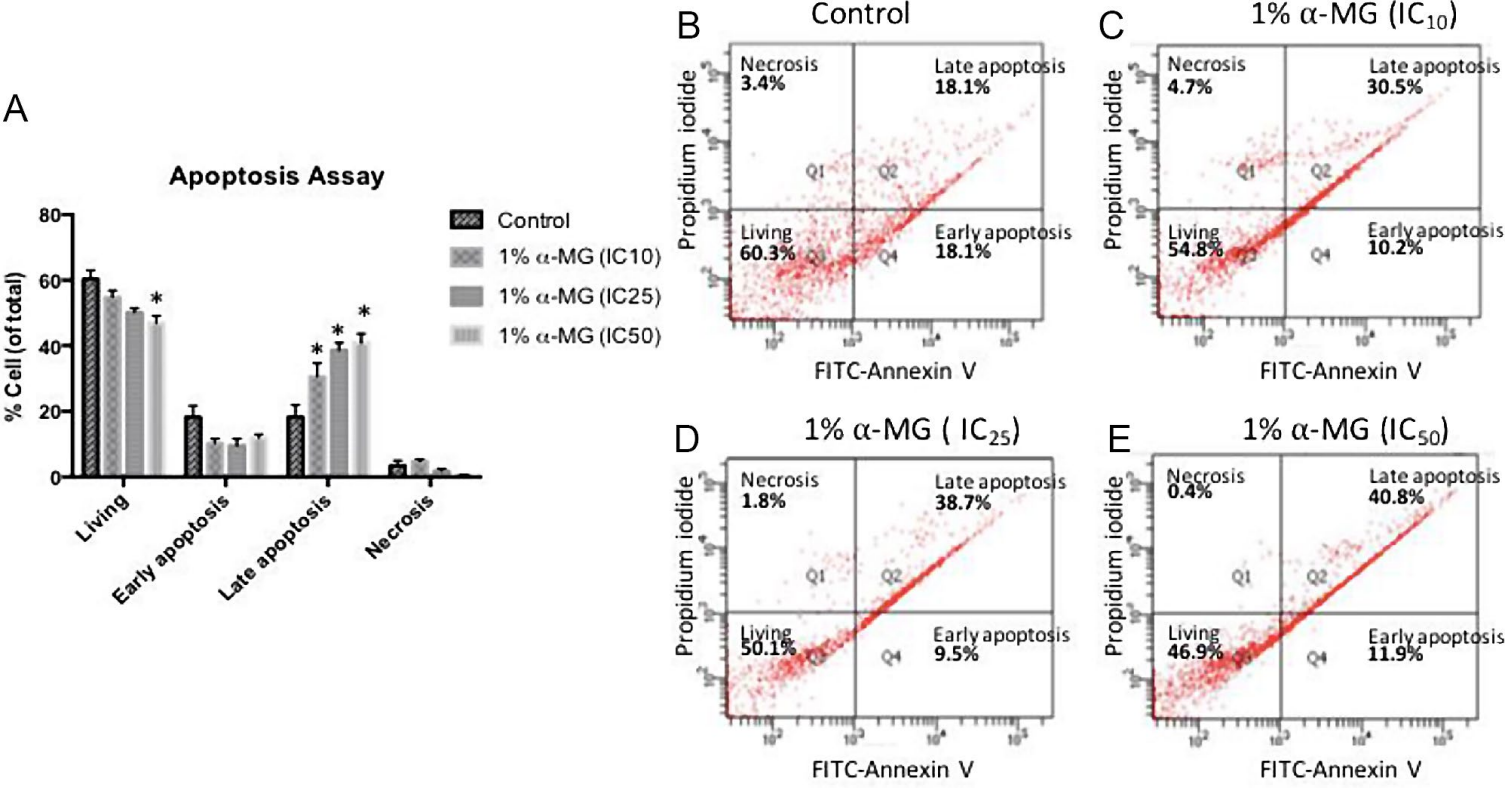
Apoptosis assay of SCC-25 cells exposed with the formulation of 1% α-MG in orabase gel (Formulation 2) at IC_10_, IC_25_ and IC_50_ concentrations compared to control (**A**). Flow cytometry data showed percentage of cells stained with FITC-Annexin V and Propidium iodide in four different states (living, early apoptosis, late apoptosis and necrosis) (**B**-**E**). Data were expressed as mean +/- SEM. Asterisk indicates statistically significant differences from control at p-value < 0.05

The effect of the 1% α-MG in orabase gel on apoptosis induction in SCC-25 cells was determined using flow cytometry technique. Annexin V and propidium iodide (PI) staining was used to identify cells in different states including healthy living cells, early apoptosis, late apoptosis and necrosis (Fig. 4). The viable cells are unstained, therefore, showing FITC-Annexin V^−^/PI (Fig. 4b-e). Three different concentrations of 1% α-MG in orabase gel (IC_10_, IC_25_ and IC_50_) were used as treatment conditions. The result showed that exposure of cells to the gel reduced percentage of unstained living cells in a dose-dependent manner. This decrease in cell viability was statistically significance in cells treated with IC_50_ of the gel (Fig. 4a). All three concentrations of the 1% α-MG in orabase gel significantly increased percentage of late apoptotic cells, which were positively stained with FITC-Annexin V and PI (Fig. 4a-e). The FITC-Annexin V^+^/PI^+^ staining explained a loss of plasma membrane integrity in cells exposed to the gel. However, the statistical changes in early apoptosis (FITC-Annexin V^+^/PI^−^) and necrosis (FITC-Annexin V^−^/PI^+^) were not observed in cells exposed with the gel in all conditions tested (Fig. 4a). Taken together, the 1% α-MG in orabase gel appeared to reduce cell viability by promoting late apoptosis in SCC-25 oral cancer cells.

### Antiviral activity of oral gel containing α-mangostin

#### Effects on HPV-16 pseudovirus infection

**Fig. 5:**
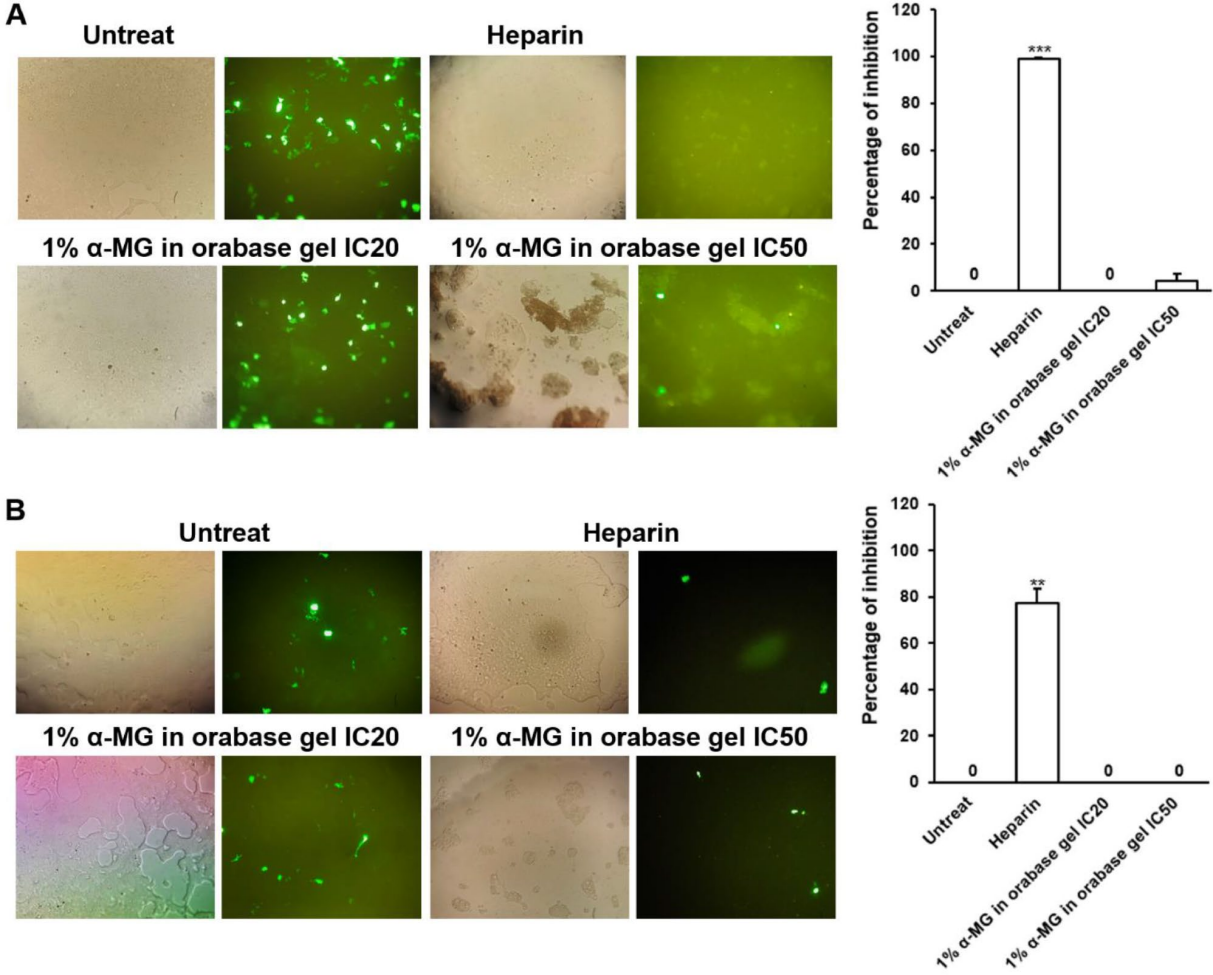
Effect of the formulation of 1% α-MG in orabase gel on HPV-16 pseudovirus infection in 293FT cell. HPV-16 pseudovirus at multiplicity of infection (MOI) at 0.05 was performed to infect 293FT cells. For pre-attachment step (**A**), the formulation of 1% α-MG in orabase gel (Formulation 2) at CC_20_ and CC_50_was mixed to pseudovirus and incubated at 37 °C for 1 h. Subsequently, the mixture was incubated with 293FT cells for 4 h and then removed and replaced with complete medium incubated for 48 h. For adsorption step (**B**), the 293FT cells were adsorbed with pseudovirus at 20 °C for 2 h. The gel was added to the cells after removal of the pseudovirus and then incubated for 48 h. Heparin (400 µM) was used as a positive control. Total cells and green fluorescent cells were counted in the same area by hemocytometer. The number of green fluorescent cells was relative to total cells and compared to untreated cells. The different percentage of inhibition was statistically assessed by an unpaired t test. The significant value was indicated as ** (*P* < 0.01) and *** (*P* < 0.001), respectively

To study effects of 1% α-MG in orabase gel on HPV-16 pseudovirus, the virus at MOI 0.05 was used to infect the cells with or without the gel treatment at the CC_20_ and CC_50_. The cytotoxic concentration at CC_20_ and CC_50_ of 293FT was 1.34 ± 0.05 mg/ml and 2.96 ± 0.07 mg/ml, respectively (Table 1). The results showed that the gel cannot inhibit HPV-16 pseudovirus infection at both pre-attachment and adsorption steps compared to Heparin as a positive control (Fig. 5). However, at CC_50_ the gel showed a trend to inhibit HPV-16 pseudovirus infection (4.33% ± 2.96).

**Table 1:** Cytotoxicity of formulation of 1% *α*-MG in orabase gel in 293FT cell line

Cell	Cytotoxic concentration at 20% (CC_20_)	Cytotoxic concentration at 50% (CC_50_)
293FT	1.34 ± 0.05 mg/ml	2.96 ± 0.07 mg/ml

### Antimicrobial activities of oral gel containing α-mangostin

The minimal growth inhibition concentration and the minimal bactericidal concentration of the α-MG compound and 1% α-MG in orabase gel were determined. The compound showed strong antimicrobial activity against *S. mutans*, *P. gingivalis*, and *C. albicans*. However, no antimicrobial activity of the gel against these microorganisms was detected (Table 2).

**Table 2:** Antimicrobial tests of the formulation of 1% *α*-MG in orabase gel

Samples	Minimum Inhibitory Concentrations (MIC)		
	*S. mutans* ATCC 25175	*P.gingivalis* ATCC 33277	*C. albican*s ATCC 90028
*α*-MG compound	10 (µg/ml)	10 (µg/ml)	10 (µg/ml)
1% *α*-MG in orabase gel	> 1000(µg/ml)	> 1000 (µg/ml)	> 1000 (µg/ml)
Blank	>1000 (µg/ml)	> 1000 (µg/ml)	> 1000 (µg/ml)
Vancomycin	1 (µg/ml)		
Amphotericin B			16 (µg/ml)
Metronidazole		8 (µg/ml)	

### NO inhibitory activity of oral gel containing α-mangostin

**Fig. 6:**
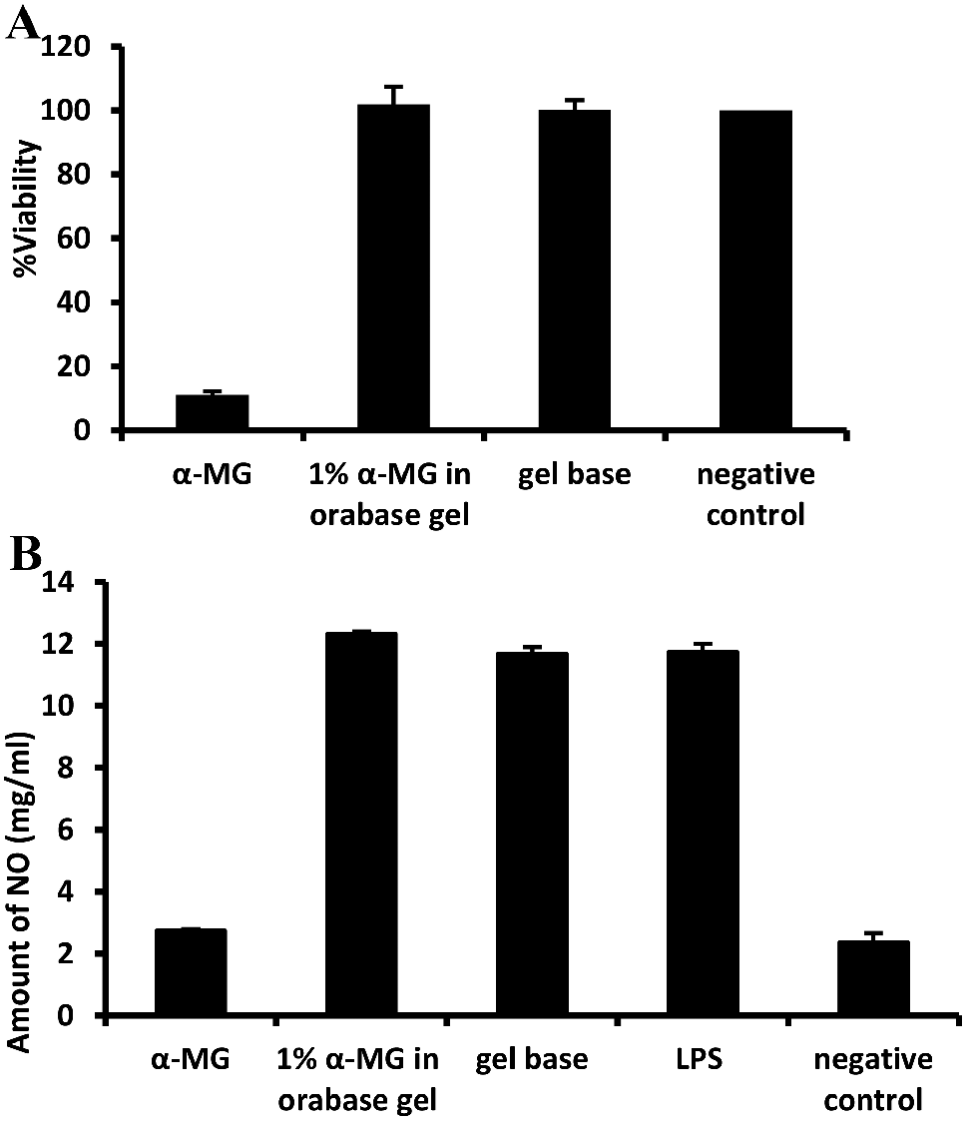
Cytotoxicity of α-MG compound and the formulation of 1% α-MG in orabase gel (Formulation 2) on murine macrophage cell line (RAW 264.7 cells). Percentage of viability of RAW 264.7 cells treated with samples concentration at 1 mg/ml (**A**) and the levels of NO produced by RAW cells (**B**) (mean ± s.d, *n* = 3)

In order to determine anti-inflammatory activity, the viability of RAW264.7 cell was assessed when exposed with the compound of α-MG and the formulation of 1% α-MG in orabase gel as well as percent inhibition of NO in the presence of the compound and the gel. The compound showed NO inhibitory activity, whereas the formulation did not demonstrate such effect (Fig. 6).

### Effects of oral gel containing α-mangostin on wound healing

**Fig. 7:**
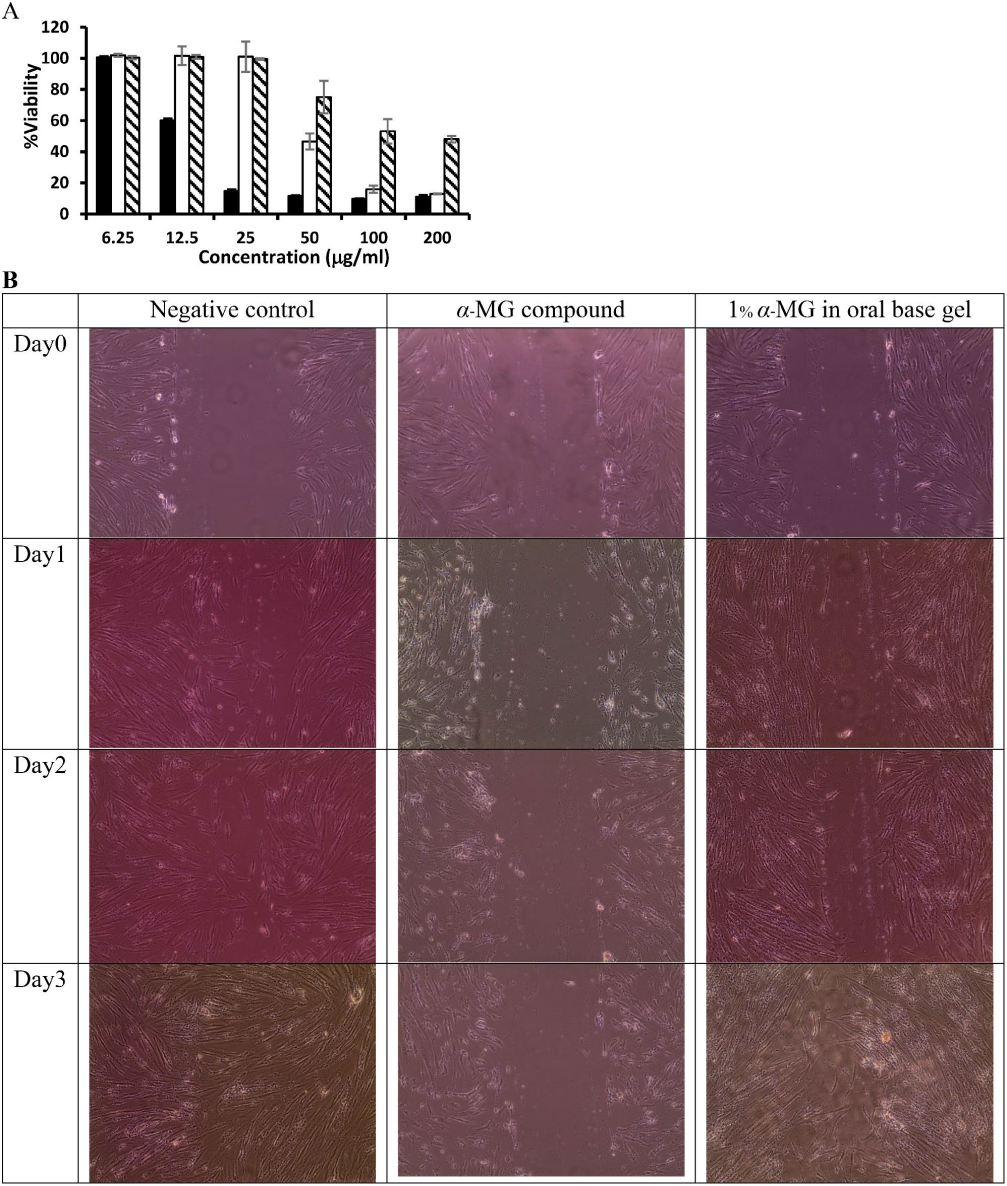
Viability and migration of human gingival fibroblast (HGF) cell line in response to α-MG compound and 1% α-MG in orabase gel. Percentage of viability of HGF cell line treated with different concentrations of α-MG compound (black bar) and the formulation of 1% α-MG in orabase gel (white bar) in comparison with blank orabase gel (strip bar) (mean ± s.d, *n* = 4) (**A**). Migration of human gingival fibroblast (HGF) after having been scratched and treated with the samples of α-MG compound and the formulation of 1% α-MG in orabase gel at day 0, 1 and 2 (**B**): left panel is Negative control; middle panel is α-MG compound and right panel is the Formulation of 1% α-MG in orabase gel

Cytotoxic effect of the compound and the 1% α-MG orabase gel was also determined in HGF (Fig. 7A). The cytotoxic effects were observed when the concentration of the compound was 12.5 µg/ml (IC_50_ of 13.65 µg/ml) and the concentration of the compound in the gel was 50 µg/ml (IC_50_ of 80.50 µg/ml). Measurement of cell migration was determined in vitro by scratch assay. A HGF layer was subjected to scratch and treated with the α-MG compound and the formulation of 1% α-MG in orabase gel (1 mg/ml) at Day 1, Day 2 and Day3 after incubation. The effect of the compound and the gel in the in vitro scratch assay using HGF is shown in Table 3 (*n* = 3). It was noted that the cell morphology was changed in the presence of the compound, and the gel did not significantly increase the cell migration even at Day3 (Fig. 7B).

**Table 3:** Effect of *α*-MG compound and 1% *α*-MG in orabase gel on in vitro scratch assay using human gingival fibroblast (*n* = 3)

Days	%Migration rate cells (mean ±sd)		
	*α*-MG compound	1% *α*-MG in orabase gel	Negative control
0	0.00± 0.00	0.00± 0.00	0.00± 0.00
1	5.46 ± 3.20	30.07 ± 1.99	28.84 ± 1.60
2	23.46 ± 0.68	51.46 ± 1.10	78.73 ± 1.24
3	35.78 ± 2.11	90.38 ± 3.50	99.85 ± 0.44

## Discussion

This study demonstrated that the compound from mangosteen peel extract containing α-MG can inhibit OSCC cell line SCC-25. The formulation of 1% α-MG in orabase gel inhibited the growth and viability of the oral cancer cell line by inducing apoptosis. We found that varying percentage of α-MG in the extract affected the viability of SCC-25. Different formulations of α-MG oral gel influenced the cell viability. The induction of apoptotic activity on the cancer cell by 1% α-MG in orabase gel was dose dependent with pronounced effect in late apoptosis. Both α-MG compound and the α-MG oral gel formulation appeared to reduce cell viability of OKC. Also, 1% α-MG in orabase gel seemed to inhibit HPV-16 pseudovirus infection at the attachment step. The α-MG compound but not the formulation showed antimicrobial activities against *S. mutans, P gingivalis* and *C. albicans*. No significant NO inhibitory activity and no enhancing on wound healing effects were observed in response to both the compound and the formulation.

In the present study, Mangosteen peel extract containing *α*-MG demonstrated anticancer effects in SCC-25 cell line. This finding of anticancer activity of α-MG in human OSCC cell line is consistent with those reported in the literature [3, 14]. A study by Kwak et al. [3] revealed that *α*-MG inhibited proliferation and induced cell death in OSCC cells in a dose- and time-dependent manner. A recent study by Tangsuksan et al. [14] demonstrated cytotoxic effect of mucoadhesive film containing α-MG on OSCC cell line. In the present study, we also found that percentage of α-MG in the extract affected anticancer activity of the compound.

Apoptosis is the death program regulation to eliminate damaged cells and maintain cellular homeostasis [18]. The process occurs via two major pathways; the extrinsic pathway activated by death receptor stimulation and the intrinsic pathway dominated by mitochondria in response to DNA damage and other stimuli [18]. Several changes can be seen in apoptotic cells including membrane blebbing, increase cellular permeability, nuclear condensation and DNA fragmentation [19].

In the present study, *α*-MG compound demonstrated anticancer effects in the human OSCC cell line SCC-25. This finding is in consistent with that reported in the literature [20]. The anticancer activity of *α*-MG was noted through the inhibition of cell growth and the promotion of apoptosis. A previous study by Kwak et al. [3] using Hoechst staining and flow cytometry with annexin V and PI staining reported that *α*-MG induced apoptosis in human OSCC cells. In human leukemia cells, *α*-MG was also shown to induce apoptosis via mitochondrial dysfunction [21]. In addition, *α*-MG acts as an effective anticancer agent against pancreatic tumors in vitro and in vivo [22]. In our study, the formulation of 1% *α*-MG in orabase gel also showed apoptotic effects on SCC-25. However, molecular mechanisms underlying the anticancer effects of the formulation in human OSCC are not well established and need further investigations.

It is well accepted that regulation of cell cycle is crucial to the cell survival, proliferation, and differentiation. Dysregulation of the cell cycle may lead to carcinogenesis [23]. A previous study by Kwak et al. [3] demonstrated that *α*-MG treatment caused an accumulation of cells in the G1 phase. In addition, *α*-MG downregulated the expression of the cyclin/CDK complexes, which are controllers of the cell cycle in a time-dependent manner [3]. Thus, further study should be performed to determine if the formulation of 1% *α*-MG in orabase gel can inhibit carcinogenesis through the cell cycle dysregulation. Anticancer effects of the formulation on chemical induced OSCC should also be conducted in an animal model. In addition, clinical trial should be carried out to confirm the findings.

In the present study, it should be noted that the two formulations of oral gel containing *α*-MG at the concentration that can inhibit the growth of SCC-25 appeared to affect viability of OKC, but less effects on HGF and RAW264.7cells. A previous study by Kwak et al. [3] reported that *α*-MG inhibited cell proliferation and induced cell death in OSCC cells with little to no effect on normal human periodontal ligament fibroblasts (PDLF) [3]. No toxicity on HGF was observed at < 1 µg/ml of α-MG, but a reduced number of viable cells was detected at a high dose of 1.5 and 2 µg/ml. α-MG showed no to minor damage to viability of RAW264.7 cells, whereas mechanical stiffness, which is involved in cell adhesion and is a critical tool for invasion of cancer, was found reduced on the cells after treatment with α-MG [11].

Although a previous study reported that α-MG has selective binding affinity to cancerous cells more than non-cancerous cells due to cell type-dependent drug activity [24], the application of 1% *α*-MG in orabase gel as a topical agent for prevention and/or treatment of OSCC must be cautious of the effects on OKC cell growth. Further studies should be performed to modify the formulation and determine if the inhibition of OSCC still remains while the effects on viability of OKC is minimized or avoided. Additional studies of topical application of the 1% *α*-MG in orabase gel on chemical induced oral cancer in an animal model should be conducted.

Our study demonstrated that the 1% α-MG in orabase gel formulation seemed to inhibit HPV-16 pseudovirus infection at attachment step. This study is consistent with our recent findings reported by Tangsuksan et al. [14]. Thus, the gel may be useful to inhibit HPV-16 infection associated OSCC. A previous study reported that α-MG possesses antiviral activity as it inhibits dengue virus in infected HepG2 cell lines [25]. Furthermore, the expression of various chemokines and cytokines were significantly suppressed when treated with α-MG [25], suggesting that the compound may affect the inflammatory response upon infection.

In the present study, the formulation of 1% α-MG in orabase gel neither showed NO inhibitory activity nor enhanced wound healing in HGF. In contrast, our previous study reported that mucoadhesive film containing α-MG showed potent anti-inflammatory and enhanced wound healing [14]. This may indicate that different components in the formulation may have effects or interferences on the release of the active ingredients resulting in no NO inhibitory activity and no wound healing effects of the gel. A previous study also reported that the Mangosteen peel extract can reduce inflammation related to gingivitis in rats [26]. Kresnoadi et al. [27] further demonstrated that the inflammation of post-tooth extraction in guinea pigs was reduced by the total extract of Mangosteen pericarp. This may be due to the extract ability in reducing the expression of nuclear factor kβ (NfkB) and receptor activator of nuclear factor-kβ ligand [27]. These reports encourage the use of Mangosteen peel extract in promoting oral health. However, it should be noted that different formulations of α-MG may have different effects and/or different bioactivities.

Mangosteen peel extracts have been demonstrated to prevent oral diseases and promote oral health by inhibiting common oral pathogens [14]. In the present study, the α-MG compound showed antimicrobial activity against *S. mutans*, *P. gingivalis* and *C. albicans*. However, no antimicrobial activity against the microorganisms was noted with respect to the formulation of 1% α-MG in orabase gel. In contrast, our previous study reported that oral spray containing α-MG and lawsone methyl ether (LME) showed potent antimicrobial activity against those common oral pathogens [28]. A previous study by Phunpee et al. [29] revealed that a-MG grafted with chitosan can inhibit *S. mutans* ATCC 25177 and *C. albicans* ATCC 10231 growth with MIC values of 6.4 and 25.6 mg/mL, respectively. A study by Pribadi et al. [30] also reported that the Mangosteen pericarp in ethanol extract can inhibit glucosyltransferase enzyme which is essential forcaries progression. Again, this may indicate that different components in the formulation may differentially affect the release of the active ingredients of the oral products resulting in no antimicrobial activity of 1% α-MG in orabase gel against those common oral pathogens in the present study.

There are some limitations in drug delivery of α-MG in oral cancer prevention and treatment including thickness of keratinization tissues of the oral mucosa, which may influence drug permeability into the OPMD lesions. The environment in the oral cavity is also a critical factor that may affect drug dissolution, release, and permeation, especially by saliva and oral tissues. Saliva secretion can dilute and shear active agents from targeting tissues, making liquid and semi-solid formulations difficult to stabilize for local delivery. Movements of the tongue, which incorporates secretions, may also eliminate drug from applying location. Thus, further studies should be focused on nanotechnology with a sustained release system and appropriate carrier system of the active compound.

In conclusion, the two formulations of oral gel containing α-MG showed an effective anticancer activity against OSCC cell line SCC-25. The formulation of 1% α-MG in orabase gel induced apoptosis in the tumor cells. 1% α-MG in orabase gel demonstrated anticancer activity by inducing apoptosis although low level of cytotoxicity observed in OKC was present. Thus, a sustained release and appropriate carrier of novel nano-particles used in a drug delivery system of α-MG that specifically targets only the cancer cells should be investigated. Experimenting on development of drug dosage, system, and efficacy alongside operating in vivo and clinical trials should also be performed. Effects of the gel containing α-MG on other oncogenic viruses such as Epstein Barr virus (EBV) and human immunodeficiency virus (HIV), which are risk factors for oral cancers, are not well established and need further investigation.

## Data Availability

Data and material of this study are available upon request to the first author via email.

## Abbreviations

*α*-MG: *α*-mangostin
DMEM: Dulbecco’s Modified Eagles Medium
DMSO: Dimethyl sulfoxide
EBV: Epstein Barr virus
FBS: Fetal bovine serum
HEK: Human embryonic kidney
HGF: Human gingival fibroblast
HIV: Human immunodeficiency virus
HPV: Human papillomavirus
LME: Lawsone methyl ether
LPS: Lipopolysaccharide
MTT: 3-(4,5-dimethylthiazol-2-yl)-2,5-diphenyltetrazolium bromide
MOI: Multiplicity of infection
NO: Nitric oxide
OD: Optical density
OKC: Oral keratinocytes
OPMD: Oral potentially malignant disorders
OSCC: Oral squamous cell carcinoma
PEG: Polyethylene glycol
MIC: Minimal growth inhibition concentration
MBC: Minimal bactericidal concentration
PDLF: Periodontal ligament fibroblasts
SCC: Squamous carcinoma cell
